# Generation of an In Vitro Cartilage Aging Model Using Human Sera from Old Donors

**DOI:** 10.3390/bioengineering12080823

**Published:** 2025-07-30

**Authors:** Sophie Hines, Meagan J. Makarczyk, Joseph Garzia, Hang Lin

**Affiliations:** 1 Department of Orthopaedic Surgery, School of Medicine, University of Pittsburgh, Pittsburgh, PA 15217, USA; soh37@pitt.edu (S.H.); mjm392@pitt.edu (M.J.M.); jag528@pitt.edu (J.G.); 2 Department of Bioengineering, Swanson School of Engineering, University of Pittsburgh, Pittsburgh, PA 15217, USA; 3 Orland Bethel Family Musculoskeletal Research Center (BMRC), School of Medicine, University of Pittsburgh, Pittsburgh, PA 15217, USA

**Keywords:** serum, aging, chondrocyte, cartilage, matrix metalloproteinase

## Abstract

Cartilage degradation is a key feature of osteoarthritis (OA), a joint disease that significantly impacts the quality of life of the elderly population. While advanced age is recognized as one of the major risk factors for OA, the underlying mechanisms are not fully understood. Research involving cartilage from aged animals has improved our understanding of the changes associated with aging. However, studies with aged animals can be time-consuming and costly. In this study, we investigate the use of human sera from older donors as a stressor to induce aging-like changes in cultured human chondrocytes. First, we assess the expression levels of markers related to chondrogenesis, hypertrophy, fibrosis, and inflammation in human chondrocytes treated with sera from younger or older human donors. Next, we evaluate the regenerative potential of these sera-treated chondrocytes by stimulating them with the anabolic factor transforming growth factor (TGF)-β3. The results show that treatment with sera from older donors induced an aging-like phenotype in chondrocytes and impaired their ability to generate new cartilage. These findings provide insight into the role of systemic factors (serum) in cartilage aging and offer a novel in vitro model for studying age-related changes in chondrocytes.

## 1. Introduction

OA is a degenerative joint disease marked by cartilage degradation, synovial inflammation, subchondral bone remodeling, pain, and loss of joint function, significantly impacting quality of life [1]. The prevalence of OA increases markedly with age, as 88% of people suffering from OA are aged 45 or older, and 43% of these patients are 65 or older [2]. As the global population continues to age, understanding the interplay between age-related changes and OA pathogenesis is crucial for developing effective strategies for prevention and treatment.

During aging, chondrocytes, the primary cell type within the articular cartilage, exhibit several molecular and cellular changes that contribute to their functional decline, such as stress-induced cellular senescence, mitochondrial dysfunction, and cytoskeletal reorganization [3]. In addition, aged chondrocytes undergo functional and phenotypic changes, including reduced synthesis of extracellular matrix components, impaired proliferation potential, and increased production of proinflammatory cytokines [4,5,6]. For example, aggrecan and collagen type 2 genes are downregulated, while expression levels of interleukin-6 and 8 increased [7,8]. There are numerous factors that contribute to these physiological shifts, including the generation of DNA damage, accumulation of advanced glycation end products, and epigenetic alterations [9,10,11]. To study the mechanisms of age-related changes resulting in OA, various rodent models have been employed, including spontaneous development of OA in aged rodents, genetically modified mice to study modifications of matrix components to induce an OA phenotype during aging, and recently, heterochronic parabiosis models [12]. While animal models play a critical role in studying aging and OA pathogenesis, they can be time-consuming and costly. There are also ethical concerns with animal research [13]. In vitro cultures can potentially overcome these limitations, but there currently is a paucity of in vitro aging models. For example, using primary chondrocytes isolated from the articular cartilage of older donors is the most straightforward strategy, but native chondrocytes have limited expansion capacity and tend to dedifferentiate in vitro [14]. Therefore, stem cells that have chondrogenic potential have been extensively studied as alternative sources of chondrocytes, such as mesenchymal stromal cells (MSCs) and induced pluripotent stem cells [15].

Currently, several methods have been tested to induce aging-like changes in chondrocytes, such as hydrogen peroxide or chemotherapeutic agents [16,17]. However, a physiologically relevant method to induce chondrocyte aging in vitro has not been fully established. In recent years, it has been shown that systemic factors can contribute to age-related changes. Specifically, heterochronic parabiosis models, the surgical pairing of young and old mice, have indicated that shared systemic circulation from old animals can impact the cartilage health of young animals [18,19,20]. Additionally, the use of sera to induce age-related changes in vitro in various cell types has been recently explored [21,22,23,24]. In this study, it was thus hypothesized that human sera from older donors could be used to induce age-related changes in human-MSC-derived chondrocytes. We analyzed changes in gene expression from cartilage constructs treated with the older sera group and compared the results to cartilage constructs from the group treated with younger sera. We also examined the differences in chondrogenic potential by stimulating cartilage constructs that were treated with sera from younger or older donors with transforming growth factor (TGF)-β3 to observe changes in chondrogenic potential between the groups.

## 2. Materials and Methods

### 2.1. Isolation and Expansion of Human MSCs (hMSCs)

Human bone marrow-derived MSCs were isolated from surgical waste of total joint arthroplasty with IRB approval (University of Washington and University of Pittsburgh) following the previously established protocol [25]. To obtain sufficient cell numbers, hMSCs were pooled from 12 different donors (6 male and 6 female) from ages ranging from 25 to 84 years, with specific information found in Table 1. hBMSCs were expanded in growth medium (GM; Dulbecco’s Modified Eagle Medium (DMEM, high glucose; Gibco, Grand Island, NY, USA), 1× antibiotic/antimycotic (ibco), 10% fetal bovine serum (FBS; Gemini Bio-Products, West Sacramento, CA, USA)) supplemented with 1ng/mL basic fibroblast growth factor (bFGF; Ray Biotech, Norcross, GA, USA). Trypsin was used to detach hBMSCs (trypsin-0.25% ethylenediaminetetraacetic acid; Thermo Fisher, Waltham, MA, USA) once they reached 80–90% confluence. This was repeated until hBMSCs reached passage 5, which were used for all studies below.

### 2.2. Preparation of Gelatin Methacryloyl (GelMA)

GelMA was made based on a previously established method [26]. Briefly, 17 g of gelatin type B from bovine skin (Sigma-Aldrich, St. Louis, MO, USA) was added to 500 mL of distilled water and placed on a 37 °C shaker at 100 rpm for 30 min. Once gelatin powder was completely dissolved, 13 mL of methacrylic anhydride (Sigma-Aldrich) was added. The solution was kept overnight at 150 rpm at 37 °C to allow reaction. The mixture was then aliquoted into 70 mL dialysis bags (Thermo Fisher) and dialyzed in water for 5 days to purify GelMA. After lyophilization, dried GelMA was weighed, and the volume of Hank’s Balanced Salt Solution (HBSS, GE Healthcare Life Sciences, South Logan, UT, USA) was added for a GelMA solution with a final concentration of 15% (*w*/*v*). To reach a p.H. of ~7.4, 1 M sodium hydroxide (NaOH, Sigma-Aldrich) was incrementally added. 1% *w*/*v* antibiotic/antimycotic was added to decrease risk of contamination. To allow for photocrosslinking, 0.15% Lithium phenyl-2,4,6-trimethylbenzoylphosphinate (LAP) (Sigma-Aldrich) was added.

### 2.3. Differentiation of hMSCs

The cell/GelMA volume ratio used was 20 million cells/mL, as established by a prior study [27]. The GelMA cell suspension was pipetted into sterile molds and cured for 2 min with 395 nm visible light illumination. Once hMSCs were encapsulated in the scaffolds, the constructs were left in GM overnight. The culture medium was switched to chondrogenic medium (CM; DMEM, 1× antibiotic/antimycotic, 1% *v*/*v* Insulin-transferrin-selenium-ethonlamine (ITS), 10^−7^ M dexamethasone, 40 µg/mL L-proline (Sigma-Aldrich), supplemented with 10 ng/mL TGF-β3 (Peprotech, Rocky Hill, NJ, USA) and 50 µg/mL ascorbic acid-2-phosphate (Sigma-Aldrich)). Media changes were performed every 2–3 days for 28 days. TGF- β3 (10 ng/mL) stimulation post serum treatment occurred for seven days, with media changes occurring every other day.

### 2.4. Human Serum Treatment and Chondrogenesis

**Figure 1:**
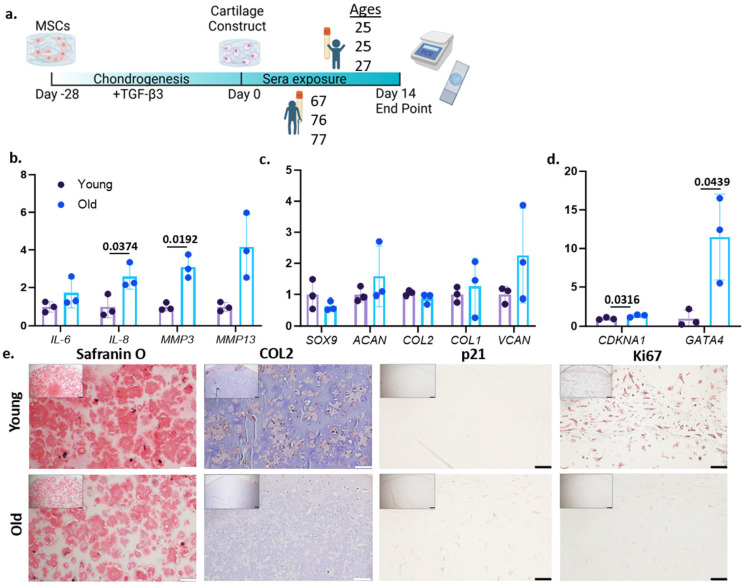
(**a**) Schematic describing experimental setup. Real-time PCR was used to measure the relative gene expression of (**b**) inflammatory genes, interleukin (IL-6), IL-8, matrix metalloproteinase (MMP)-3, and MMP13. (**c**) Chondrogenic genes, SRY-Box transcription factor (SOX)9, aggrecan (ACAN), and collagen type 2 (COL2), COL1, and versican (VCAN). (**d**) CDKN1A and GATA4. Data were normalized to the mean of the younger-sera-treated group (set as 1). Unpaired *t*-test with Welch’s Correction was used for statistical analysis, and *p*-values are presented over the bars. n = 3 biological replicates per group. (**e**) Safranin O staining and COL2 IHC (Scalebar = 100 μm), p21 and Ki67 IHC (Scalebar = 50 μm).

**Figure 2:**
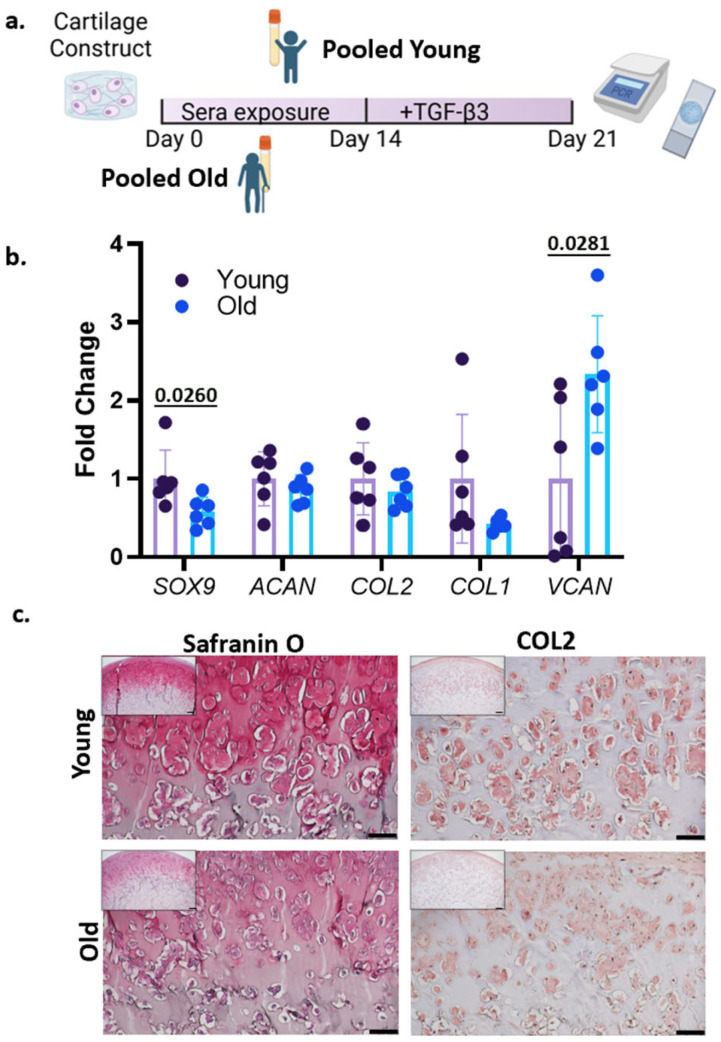
(**a**) Schematic describing experimental setup. (**b**) Real-time PCR was used to measure the relative gene expression of SOX9, ACAN, COL2, COL1, and VCAN. Data were normalized to the mean of the younger-sera-treated group (set as 1). n = 6 technical replicates per group. Unpaired *t*-test with Welch’s Correction was used for statistical analysis, and *p*-values are presented over the bars. (**c**) Safranin O staining COL2 IHC (Scale bar = 100 μm).

All human serum was purchased through ZenBios (Durham, NC, USA). Human serum was incorporated into the growth medium either from individual donors (Figure 1) or with equal portions from each donor for pooled serum (Figure 2) (DMEM, 1× antibiotic/antimycotic, 5% human serum). Donor information can be found in Table 2. After treatment, the samples were harvested for analysis or subjected to chondrogenesis for another 7 days using the conditions described in Section 2.3.

### 2.5. Total RNA Isolation and Real-Time Quantitative Reverse Transcription PCR (qRT-PCR)

QIAzol Lysis Reagent (Qiagen, Germantown, MD, USA) was utilized to isolate total RNA. RNA extraction was performed with an RNeasy Plus Universal Kit (Qiagen), followed by reverse transcription with SuperScript^®^ VILO^TM^ cDNA Synthesis Kit (Invitrogen, Carlsbad, CA, USA). Total RNA concentration was measured with a nanodrop 2000c Spectrophotomere (Thermo Fisher Scientific, Waltham, MA, USA). Quantitative real-time polymerase chain reaction (qRT-PCR) was performed on a Quantstudio 5 system (Thermo Fisher Scientific) with PowerUP SYBR Green Master Mix (Applied Biosystems, Foster City, CA, USA). Calculation of relative gene expression was performed using the comparative (^ΔΔ^Ct) method and normalized to the gene expression of ribosomal protein L13a (RPL13a) and further normalized to the younger-sera-treated groups. The sequences of primers used are summarized in Table S1.

### 2.6. Histology

10% neutral buffered formalin (Thermo Fisher Scientific) was used to fix cartilage constructs overnight at 4 °C, followed by dehydration using an increasing series of ethanol concentrations from 30% to 100%. Xylene (Thermo Fisher Scientific) was used to clear the samples, which were then embedded in paraffin sectioned at a thickness of 6 μm. Using a microtome (Model RM 2255, Leica, Buffalo Grove, IL, USA), samples were sectioned at a thickness of 6 μm. About 4 sections from the middle of the constructs were placed on each slide, with at least two slides being stained per group. Sections were then dewaxed using Histo-Clear (National Diagnostic, Atlanta, GA, USA), rehydrated with a graded series of ethanol, and stained with 0.5% Safranin O/0.005% fast green (Sigma-Aldrich) and Hematoxylin (Sigma-Aldrich). Slides were imaged with an Olympus SZX16 (Olympus, Waltham, MA, USA).

### 2.7. Immunohistochemistry (IHC)

Sections were prepared as stated in 2.6. Slides were cleared in Histo-Clear and rehydrated using a graded series of ethanol. Antigen retrieval was performed using an IHC antigen retrieval solution (Invitrogen) at 90 °C for 20 min. The slides were incubated in 3% hydrogen peroxide at room temperature for 10 min, then blocked in 1% horse serum (PK-6200 VECTASTAIN Elite ABC HRP kit, Vector Laboratories, Newark, CA, USA) for 45 min. Slides were then incubated overnight at 4 °C with antibodies against p21 (cyclin-dependent kinase inhibitor 1A), collagen II, and Ki67 (Kiel 67) (Table S2). Rabbit IgG (Invitrogen) was used in place of a primary antibody as a negative control. Biotinylated antimouse-rabbit IgG secondary antibody (VECTASTAIN Elite ABC HRP kit, PK-6200, Vector Laboratories) was used, and VECTOR NovaRED peroxidase substrate kit (SK-4800, Vector Laboratories, Newark, CA, USA) was employed for signal visualization. Quantification was performed using ImageJ Version 1.53k.

### 2.8. Statistical Analysis

Statistical analyses were performed using GraphPad Prism 9. Data were presented in the form of a histogram with error bars illustrating the standard deviation. Prior to statistical analysis, a Shapiro–Wilk test was used to assess normality. An unpaired Student’s *t*-test or a non-parametic equivalent was used for comparison between the two groups. *p* < 0.05 was the threshold to be considered statistically significant.

## 3. Results

### 3.1. Treatment of Older Sera Increases Aging-Associated Phenotypes in Engineered Cartilage

hMSCs were first encapsulated in 15% GelMA and underwent a 28-day chondrogenic culture to form cartilage tissues (Figure 1a). Successful chondrogenesis of hMSCs was confirmed with qRT-PCR and histology staining (Figure S1). We selected 14 days of sera treatment based on a preliminary test with different treatment durations (Figure S2). Specifically, we were able to see robust changes between younger and older sera treatment groups on day 14, which was also more efficient when compared to day 28 and day 56 by reducing the culture time and use of human sera. The drop off in any trends by day 56 is likely due to cell viability from prolonged culture time. In addition, we observed stronger trends and more consistent results in the constructs that were cultured with female sera compared to male sera (Figure S3), resulting in the use of only female donors for this study. All sera used for these studies are listed in Table 2.

After 14 days of treatment from three individual serum donors for both the younger and older groups, increased expression levels of interleukin (*IL*)*-6* and matrix metalloproteinase (*MMP13*) were observed in the older sera group. Statistically significant increases in *IL-8* and matrix metalloproteinase (*MMP*)*-3* were observed in the group treated with older sera (Figure 1b), which is consistent with findings in aged chondrocytes. Surprisingly, there appeared to be little effect of sera treatment on the gene expression of aggrecan (*ACAN*), collagen type 2-a1 (*COL2*), and SRY-Box transcription factor (*SOX9*) (Figure 1c). However, Safranin O staining and COL2 IHC showed less cartilage matrix in the older sera group, specifically the younger group had 18.36% COL2+ compared to only 2% COL2+ in the aged-sera-treated group (Figure 1e), which was likely due to the increased inflammation in the older-sera-treated constructs (Figure 1b). Interestingly, there was an increase in versican (*VCAN*) in the older sera treatment group (Figure 1c). This increase can be partially attributed to the increased inflammatory cytokine production, as VCAN is usually a marker for early ECM disruption [28].

Previously, we have shown that old chondrocytes display a reduced proliferation capacity compared to young cells [14]. We thus assessed the expression of cell cycle regulator p21. The results indicated a statistically significant increase in the expression of cyclin-dependent kinase inhibitor 1A (*CDKN1A*, gene of p21) in the older-sera-treated group (Figure 1d), with p21 IHC further corroborating these results (Figure 1e). Additionally, a statistically significant increase in *GATA4*, a protein related to chondrocyte aging and senescence [29], was observed in the older-sera-treated groups (Figure 1d). Lastly, the constructs from the older-sera-treated group contained fewer Ki67-positive cells than those in the younger sera group (Figure 1e), suggesting a lower proliferation potential.

### 3.2. Treatment with Human Sera and TGF-β3

Aging chondrocytes normally experience reduced chondrogenic capabilities [8]. To examine the cartilage-forming potential of in vitro “aged” chondrocytes, chondrogenic medium was added into the cultures after 14 days of pooled serum treatment (Figure 2a). After seven days, statistically significant lower levels of *SOX9*, but not *COL1*, *COL2*, or *ACAN*, were observed in the constructs pretreated by older sera compared to those treated with younger sera (Figure 2b). Interestingly, *VCAN* expression was increased in the older group. Safranin O staining and COL2 IHC showed that the cartilage matrix was less prominent in the older-sera-treated group, accounting for 16.18% compared to 31.77% in the younger-sera-treated group, further demonstrating the decreased chondrogenic potential of chondrocytes treated by older sera (Figure 2c).

## 4. Discussion

OA is a complex, degenerative joint disease that predominantly affects older individuals, and its pathogenesis involves a combination of age-related changes in the joint tissues and environmental factors. The aim of this study was to develop an in vitro model to stimulate age-related changes in human cartilage tissue. Our results indicate that aging-related changes in the systemic environment can influence cartilage health and chondrogenic capabilities, providing new insights into the mechanisms underlying OA pathogenesis. The results suggest that older human sera effectively cause the age-related inflammatory shifts in chondrocytes without significantly altering chondrogenic gene expression. Specifically, there were no significant changes in *ACAN* or *COL2* expression levels between the younger- and older-sera-treated groups. However, this was contrasted by a robust upregulation of inflammatory mediators, such as *IL-6*, *IL-8*, *MMP-3,* and *MMP-13* in older-sera-treated constructs, suggesting that catabolic processes are disproportionately activated, which is consistent with findings towards a proinflammatory phenotype known as “inflammaging”. This shift toward an inflammatory phenotype in older-sera-treated chondrocytes likely contributes to the observation of reduced ECM integrity observed in the histological staining. Despite relatively stable chondrogenic gene expression, Saf O and COL2 IHC showed diminished ECM deposition, consistent with increased proteolytic activity driven by upregulated MMPs. Additionally, we observed upregulated expression of *VCAN* in the aged-sera-treated group, which is consistent with early ECM disruption in early OA and is known to be upregulated in cases of inflammation [28]. The observed elevation of p21 and GATA4 expression further supports a phenotype that is consistent with inflammaging, rather than terminal senescence [5,30]. The Ki67 IHC suggested that there was a decline of proliferation in the aged-sera-treated group. In the future, we can harvest these cells by breaking down the scaffolds and directly test their proliferation potential. It should be noted that no significant difference was observed between the younger and older groups after 56 days of treatment (Figure S2). This may be associated with the prolonged in vitro culture masking the influence of serum age. The underlying mechanism should be investigated in the future.

Numerous heterochronic parabiosis studies have observed the effects of aged systemic milieu on young rodents. Across all studies, age-related changes can be observed in young rodents, suggesting that there are systemic factors that can accelerate aging on a cellular and tissue level and contribute to age-related pathologies [19,31,32]. Due to the widely recognized effects of the systemic milieu on the aging of various tissues, researchers have begun investigating the use of ex vivo human sera with in vitro cellular models to investigate the response to an aged environment. These studies have been found to show age-related increases in hypertrophy, decreases in cellular protein synthesis, and alterations in anabolic and catabolic processes [23,24,33]. Most commonly, these studies are looking specifically into muscle cell aging, stem cell function, or neurodegenerative diseases [23,24,34]. Current research suggests that the age-related effects that aged sera impose on the in vitro cells are multifaceted. Aged serum has been found to contain higher concentrations of inflammatory cytokines, damage-associated molecular patterns, and oxidative stress mediators that directly promote cellular senescence and dysfunction. Additionally, the aged serum has depleted protective factors compared to younger sera, which is abundant in growth factors, antioxidants, and regenerative proteins [35,36,37]. To the best of our knowledge, our current work represents the first relevant study testing human chondrocytes. Our results showed that 14 days of serum treatment was enough to observe statistically significant differences, but when left in culture for up to 56 days, the trends of the age-related changes diminished, which is thought to be due to cell viability at the end of the 56 days. In this study, we also observed stronger results with the female serum. This trend has been noted in other studies and is thought to be due to the predictable hormonal changes (menopause) that all females undergo, leading to less variability in the female aging process compared to male [38].

Compared to other in vitro models of chondrocyte aging, such as induction via oxidative stress or replicative senescence, our model demonstrates several advantages in physiological relevance. Most existing models primarily aim to induce cellular senescence through chemical stressors such as hydrogen peroxide or extended passaging [16,17,39]. While these methods are effective for generating some markers of cellular aging, they do not fully recapitulate the complex systemic influences that occur during aging in vivo. For instance, several studies focusing on cellular senescence utilize p16 expression as their key marker [16,17]. However, in our study, p16 did not exhibit significant changes, indicating a phenotype induced by aged human sera may represent a more physiologically relevant method to induce chondrocyte aging in vitro.

Although the results demonstrate the influence of sera on chondrocyte phenotype and function, this work has some limitations that can be addressed in the future. First, OA is a whole joint disease, and all cells in the knee undergo age-related changes that contribute to disease progression. Under physiological conditions, hyaline cartilage is not directly exposed to serum, although the active exchange between serum and synovial fluid highlights the potential impacts of serum on cartilage health. In the future, we will use our recently established microphysiological models to further simulate joint aging in vitro by including older sera as the physiological stressors [27]. Second, epigenetic changes such as DNA methylation and histone acetylation/deacetylation are commonly observed in aged tissues and contribute to decreased anabolism and increased catabolism within the cartilage [40]. This was not investigated in the current study, as we mainly focused on functional and transcriptional changes in chondrocytes induced by aged serum. Third, we chose to use a pool of MSCs from different donors, which provided us with sufficient cells to complete the study and also mitigated the donor-to-donor differences. The reproducibility of this study can be improved by testing individual MSCs. MSCs from other resources or primary chondrocytes can be tested in the future to further validate the findings from the current study. Lastly, qRT-PCR was mainly used to examine the changes in tissue phenotypes. Measuring protein levels would help to strengthen our conclusion and will be looked at in future studies.

Taken together, these findings underscore the importance of systemic inflammatory factors in cartilage aging and support the utility of older human serum as a physiologically relevant inducer of age-associated phenotypes in vitro. This model uniquely balances the preservation of the chondrogenic identity with the induction of inflammation and catabolism.

## Figures and Tables

**Table 1:** Donor information of MSCs.

Age	26	28	25	32	34	69	76	70	73	77	84	48
Sex	F	F	M	M	M	F	F	F	M	M	M	F

**Table 2:** Information of sera donors.

Age	Sex
24	Male
25	Female
25	Female
26	Male
27	Female
30	Male
67	Female
67	Male
73	Male
76	Female
76	Male
77	Female

Note: In main figures, only female sera were used.

## Data Availability

All data have been provided in the manuscript.

## Supplementary Materials

The following supporting information can be downloaded at the following links: https://www.mdpi.com/article/10.3390/bioengineering12080823/s1. Figure S1: Chondrogenesis of human mesenchymal stem cell (hMSC); Figure S2: Timepoint analysis for sera treatment; Figure S3: Male vs. Female Sera treatment; Figure S4: Results from study with another MSC pool. Table S1: Primer sequences for qRT-PCR; Table S2: All antibodies used in this study.

## References

[1] Shalhoub M., Anaya M., Deek S., Zaben A.H., Abdalla M.A., Jaber M.M., Koni A.A., Zyoud S.H. (2022). The impact of pain on quality of life in patients with osteoarthritis: A cross-sectional study from Palestine. BMC Musculoskelet. Disord..

[2] Losina E., Paltiel A.D., Weinstein A.M., Yelin E., Hunter D.J., Chen S.P., Klara K., Suter L.G., Solomon D.H., Burbine S.A. (2015). Lifetime medical costs of knee osteoarthritis management in the United States: Impact of extending indications for total knee arthroplasty. Arthritis Care Res..

[3] Loeser R.F. (2012). The effects of aging on the development of osteoarthritis. HSS J..

[4] Shane Anderson A., Loeser R.F. (2010). Why is osteoarthritis an age-related disease?. Best Pract. Res. Clin. Rheumatol..

[5] Rezus E., Cardoneanu A., Burlui A., Luca A., Codreanu C., Tamba B.I., Stanciu G.D., Dima N., Badescu C., Rezus C. (2019). The Link Between Inflammaging and Degenerative Joint Diseases. Int. J. Mol. Sci..

[6] Li Y., Wei X., Zhou J., Wei L. (2013). The age-related changes in cartilage and osteoarthritis. Biomed. Res. Int..

[7] Molnar V., Matisic V., Kodvanj I., Bjelica R., Jelec Z., Hudetz D., Rod E., Cukelj F., Vrdoljak T., Vidovic D. (2021). Cytokines and Chemokines Involved in Osteoarthritis Pathogenesis. Int. J. Mol. Sci..

[8] Loeser R.F. (2011). Aging and osteoarthritis. Curr. Opin. Rheumatol..

[9] Rahmati M., Nalesso G., Mobasheri A., Mozafari M. (2017). Aging and osteoarthritis: Central role of the extracellular matrix. Ageing Res. Rev..

[10] Copp M.E., Chubinskaya S., Bracey D.N., Shine J., Sessions G., Loeser R.F., Diekman B.O. (2022). Comet assay for quantification of the increased DNA damage burden in primary human chondrocytes with aging and osteoarthritis. Aging Cell.

[11] Ramasamy T.S., Yee Y.M., Khan I.M. (2021). Chondrocyte Aging: The Molecular Determinants and Therapeutic Opportunities. Front. Cell Dev. Biol..

[12] Rios J.L., Sapede D., Djouad F., Rapp A.E., Lang A., Larkin J., Ladel C., Mobasheri A. (2022). Animal Models of Osteoarthritis Part 1-Preclinical Small Animal Models: Challenges and Opportunities for Drug Development. Curr. Protoc..

[13] Rinwa P., Eriksson M., Cotgreave I., Backberg M. (2024). 3R-Refinement principles: Elevating rodent well-being and research quality. Lab. Anim. Res..

[14] Shen H., He Y., Wang N., Fritch M.R., Li X., Lin H., Tuan R.S. (2021). Enhancing the potential of aged human articular chondrocytes for high-quality cartilage regeneration. FASEB J..

[15] Lee J., Smeriglio P., Chu C.R., Bhutani N. (2017). Human iPSC-derived chondrocytes mimic juvenile chondrocyte function for the dual advantage of increased proliferation and resistance to IL-1beta. Stem Cell Res. Ther..

[16] Georget M., Defois A., Guiho R., Bon N., Allain S., Boyer C., Halgand B., Waast D., Grimandi G., Fouasson-Chailloux A. (2023). Development of a DNA damage-induced senescence model in osteoarthritic chondrocytes. Aging.

[17] Yagi M., Endo K., Komori K., Sekiya I. (2023). Comparison of the effects of oxidative and inflammatory stresses on rat chondrocyte senescence. Sci. Rep..

[18] Li L., Wei X., Geng X., Duan Z., Wang X., Li P., Wang C., Wei L. (2018). Impairment of chondrocyte proliferation after exposure of young murine cartilage to an aged systemic environment in a heterochronic parabiosis model. Swiss Med. Wkly..

[19] Gonzalez-Armenta J.L., Li N., Lee R.L., Lu B., Molina A.J.A. (2021). Heterochronic Parabiosis: Old Blood Induces Changes in Mitochondrial Structure and Function of Young Mice. J. Gerontol. Ser. A Biol. Sci. Med. Sci..

[20] Lagunas-Rangel F.A. (2024). Aging insights from heterochronic parabiosis models. npj Aging.

[21] Carson B.P., Patel B., Amigo-Benavent M., Pauk M., Kumar Gujulla S., Murphy S.M., Kiely P.A., Jakeman P.M. (2018). Regulation of muscle protein synthesis in an in vitro cell model using ex vivo human serum. Exp. Physiol..

[22] Catteau M., Gouzi F., Blervaque L., Passerieux E., Blaquiere M., Ayoub B., Bughin F., Mercier J., Hayot M., Pomies P. (2020). Effects of a human microenvironment on the differentiation of human myoblasts. Biochem. Biophys. Res. Commun..

[23] George T., Velloso C.P., Alsharidah M., Lazarus N.R., Harridge S.D. (2010). Sera from young and older humans equally sustain proliferation and differentiation of human myoblasts. Exp. Gerontol..

[24] Carlson M.E., Conboy I.M. (2007). Loss of stem cell regenerative capacity within aged niches. Aging Cell.

[25] Lin H., Yang G., Tan J., Tuan R.S. (2012). Influence of decellularized matrix derived from human mesenchymal stem cells on their proliferation, migration and multi-lineage differentiation potential. Biomaterials.

[26] Lin H., Zhang D., Alexander P.G., Yang G., Tan J., Cheng A.W., Tuan R.S. (2013). Application of visible light-based projection stereolithography for live cell-scaffold fabrication with designed architecture. Biomaterials.

[27] Li Z., Lin Z., Liu S., Yagi H., Zhang X., Yocum L., Romero-Lopez M., Rhee C., Makarcyzk M.J., Yu I. (2022). Human Mesenchymal Stem Cell-Derived Miniature Joint System for Disease Modeling and Drug Testing. Adv. Sci..

[28] Wight T.N., Kang I., Merrilees M.J. (2014). Versican and the control of inflammation. Matrix Biol..

[29] Xiong H., Hua F., Dong Y., Lin Y., Ying J., Liu J., Wang X., Zhang L., Zhang J. (2022). DNA damage response and GATA4 signaling in cellular senescence and aging-related pathology. Front. Aging Neurosci..

[30] Kang C., Xu Q., Martin T.D., Li M.Z., Demaria M., Aron L., Lu T., Yankner B.A., Campisi J., Elledge S.J. (2015). The DNA damage response induces inflammation and senescence by inhibiting autophagy of GATA4. Science.

[31] Ashapkin V.V., Kutueva L.I., Vanyushin B.F. (2020). The Effects of Parabiosis on Aging and Age-Related Diseases. Adv. Exp. Med. Biol..

[32] Lei C., Colangelo D., Patil P., Li V., Ngo K., Wang D., Dong Q., Yousefzadeh M.J., Lin H., Lee J. (2020). Influences of circulatory factors on intervertebral disc aging phenotype. Aging.

[33] Allen S.L., Marshall R.N., Edwards S.J., Lord J.M., Lavery G.G., Breen L. (2021). The effect of young and old ex vivo human serum on cellular protein synthesis and growth in an in vitro model of aging. Am. J. Physiol. Cell Physiol..

[34] Voirin A.C., Celle S., Perek N., Roche F. (2020). Sera of elderly obstructive sleep apnea patients alter blood-brain barrier integrity in vitro: A pilot study. Sci. Rep..

[35] (2025). Early prediction of healthy ageing and age-related diseases using blood protein biomarkers. Nat. Metab..

[36] Skowronska-Krawczyk D. (2023). Hallmarks of Aging: Causes and Consequences. Aging Biol..

[37] Lehallier B., Gate D., Schaum N., Nanasi T., Lee S.E., Yousef H., Moran Losada P., Berdnik D., Keller A., Verghese J. (2019). Undulating changes in human plasma proteome profiles across the lifespan. Nat. Med..

[38] Hagg S., Jylhava J. (2021). Sex differences in biological aging with a focus on human studies. elife.

[39] Kirsch V., Ramge J.M., Schoppa A., Ignatius A., Riegger J. (2022). In Vitro Characterization of Doxorubicin-Mediated Stress-Induced Premature Senescence in Human Chondrocytes. Cells.

[40] Pal S., Tyler J.K. (2016). Epigenetics and aging. Sci. Adv..

