# Supplementary material for: Generation of an In Vitro Cartilage Aging Model Using Human Sera from Old Donors
Source: Bioengineering (Basel). 2025 Jul 30;12(8):823. doi: 10.3390/bioengineering12080823 (PMC12383633; doi:10.3390/bioengineering12080823)
Supplement: Supplementary file 1 [file bioengineering-12-00823-s001.zip › bioengineering-3654348-supplementary.pdf]

Table S1. Primer sequences for qRT-PCR

Gene	Forward Primer (5’->3’)	Reverse Primer (5’->3’)
RPL13a	GCCATCGTGGCTAAACAGGTA	GTTGGTGTTCATCCGCTTGC
SOX9	GGCGGAGGAAGTCGGTGAAGAA	GCTCATGCCGGAGGAGGAGTGT
COL1	GAGGGCCAAGACGAGGACATC	CAGATCACGTCATCGCACAAC
COL2	GGATGGCTGCACGAAACATACCGG	CAAGAAGAGACCGGCCCTATG
ACAN	AGTCACACCTGAGCAGCATC	AGTTCTCAAATTGCATGGGGTGTC
VCAN	TGGAATGATGTTCCCTGCAA	AAGGTCTTGGCATTTTCTACAACAG
IL-6	ACTCACCTCTTCAGAACGAATTG	CCATCTTTGGAAGGTTCAGGTTG
IL-8	TTTTGCCAAGGAGTGCTAAAGA	AACCCTCTGCACCCAGTTTTC
MMP-3	CGGTTCCGCCTGTCTCAAG	CGCCAAAAGTGCCTGTCTT
MMP-13	ACTGAGAGGCTCCGAGAAATG	GAACCCCGCATCTTGGCTT
CDKNA1	AGTGGAATTAGCCCTCAGCA	CATGGTCCCTGGGTTCTTC
GATA4	CAGTCTACGTGCCCACACC	TCCCGCCTGGCTCCAT

*

Table S2. All antibodies used in this study.

Antibody	Company	Catalog No.	Dilution
Collagen Type II	Abcam	AB34712	1:500
Ki67 (Kiel-67)	Abcam	AB92742	1:250
p21 (cyclin-dependent kinase inhibitor 1)	Novus	NB100-1941	1:500

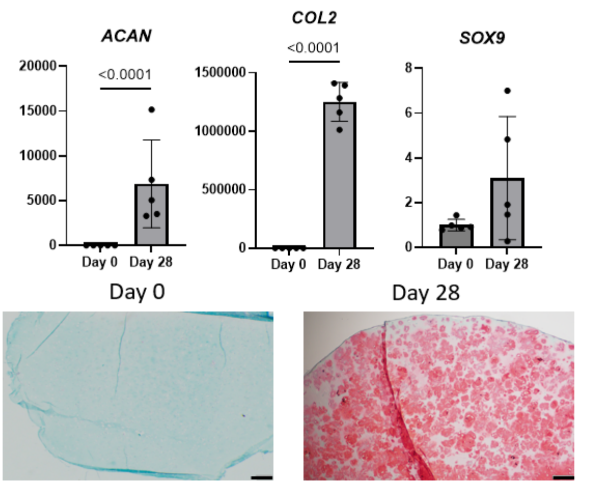

Figure S1. Chondrogenesis of human mesenchymal stem cell (hMSC) RT-qPCR analysis of relative gene expression, including *Collagen Type II (COL2), Aggrecan (ACAN),* and *SRY-Box transcription factor 9 (SOX9)*, paired T-test was performed. Safranin O/Fast green staining was used to examine matrix deposition (scale bar = 200 µm).

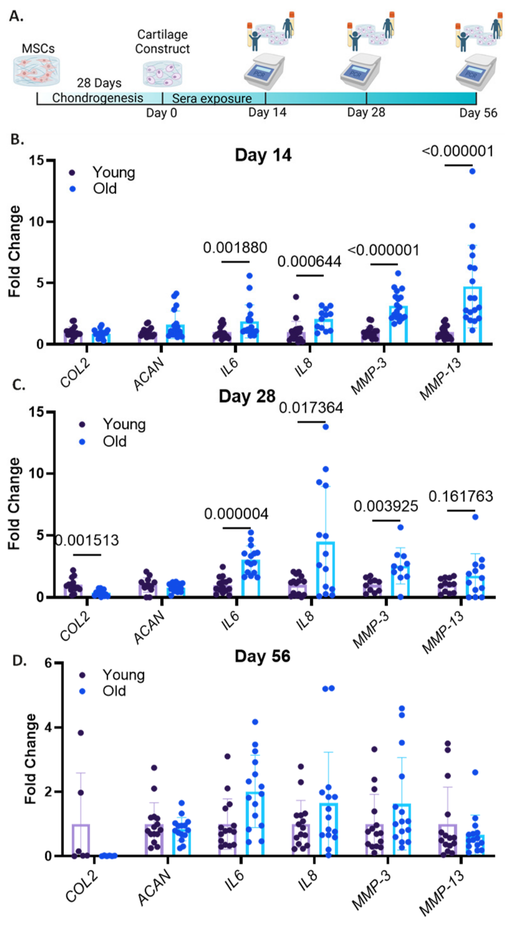

Figure S2. Timepoint analysis for sera treatment (A) Schematic illustrating experimental procedures, (B) RT-qPCR analysis of relative gene expression, including *Collagen Type II (COL2), Aggrecan (ACAN), Interleukin (IL)6, IL8, Matrix Metalloproteinase (MMP)-3,* and *MMP-13* in MSC-derived cartilage constructs treated by sera for B) 14; (C) 28; or)56 days. *Mann-Whitney test was used for all statistical analysis and significance was defined by a p-value of <0.05.*

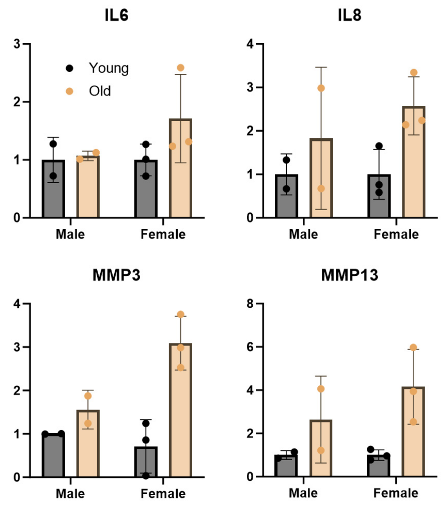

Figure S3. Male vs Female Sera treatment Relative gene expression from MSC derived chondrocyte constructs treated with human serum from young and old donors split into male and female groups of *interleukin (IL)-6, IL-8, matrix metalloproteinase (MMP)-3,* and *MMP-13. * p<0.05*

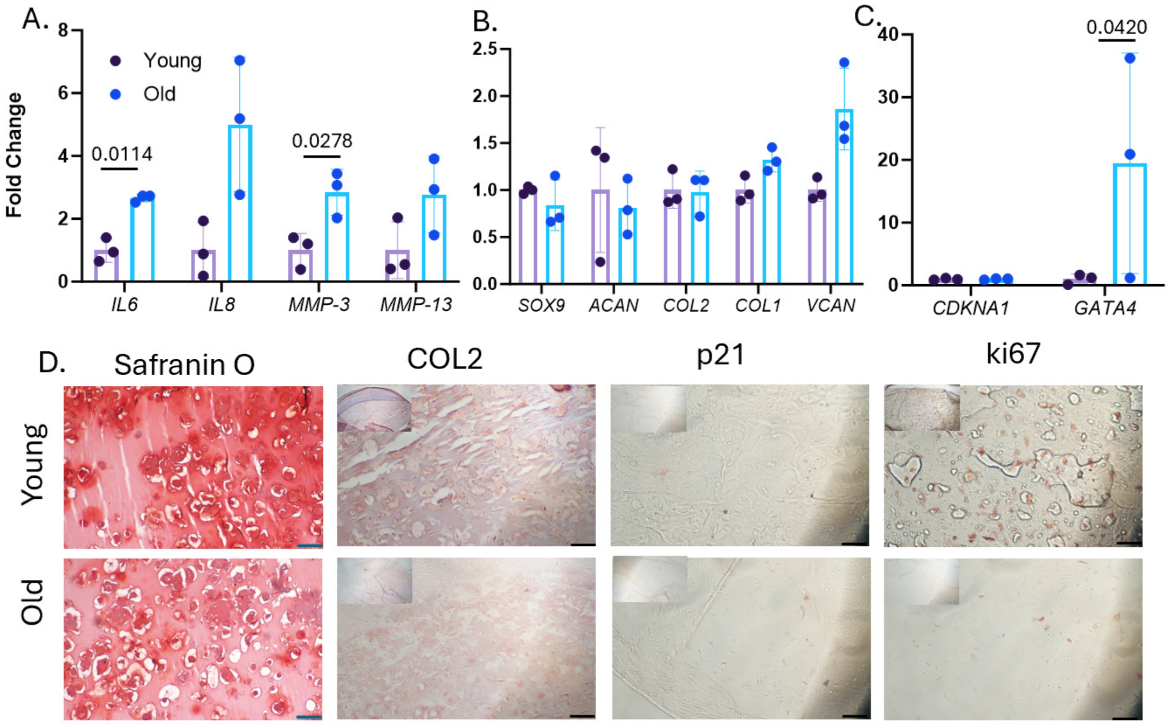

Figure S4. Validation in the second MSC pool. (A)Relative gene expression of inflammatory genes, interleukin (IL-6), IL-8, matrix metalloproteinase (MMP)– 3, and MMP-13. (B) Relative gene expression of chondrogenic genes, SRY-Box transcription factor (SOX)9, aggrecan (ACAN), and collagen type 2 (COL2), COL1, and versican (VCAN). (C)Relative gene expression of age-related genes, p21 and GATA4. (D)Safranin O staining, collagen 2 (COL2) immunohistochemistry (IHC) (Scalebar = 100 μm), p21 IHC and Ki67 IHC (Scalebar = 50 μm). The donor information was shown below.

Age	31	26	17	19	31	25	72	71	78	77	77	84
Sex	F	F	F	M	M	M	F	F	F	M	M	M
